# Identifying Mendelian disease genes with the Variant Effect Scoring Tool

**DOI:** 10.1186/1471-2164-14-S3-S3

**Published:** 2013-05-28

**Authors:** Hannah Carter, Christopher Douville, Peter D Stenson, David N Cooper, Rachel Karchin

**Affiliations:** 1 Department of Biomedical Engineering and Institute for Computational Medicine, Johns Hopkins University, 3400 N. Charles St., Baltimore, Maryland USA; 2 Institute of Medical Genetics, School of Medicine, Cardiff University, Hearth Park, Cardiff CF14 4XN, UK

## Abstract

**Background:**

Whole exome sequencing studies identify hundreds to thousands of rare protein coding variants of ambiguous significance for human health. Computational tools are needed to accelerate the identification of specific variants and genes that contribute to human disease.

**Results:**

We have developed the Variant Effect Scoring Tool (VEST), a supervised machine learning-based classifier, to prioritize rare missense variants with likely involvement in human disease. The VEST classifier training set comprised ~ 45,000 disease mutations from the latest Human Gene Mutation Database release and another ~45,000 high frequency (allele frequency *>*1%) putatively neutral missense variants from the Exome Sequencing Project. VEST outperforms some of the most popular methods for prioritizing missense variants in carefully designed holdout benchmarking experiments (VEST ROC AUC = 0.91, PolyPhen2 ROC AUC = 0.86, SIFT4.0 ROC AUC = 0.84). VEST estimates variant score p-values against a null distribution of VEST scores for neutral variants not included in the VEST training set. These p-values can be aggregated at the gene level across multiple disease exomes to rank genes for probable disease involvement. We tested the ability of an aggregate VEST gene score to identify candidate Mendelian disease genes, based on whole-exome sequencing of a small number of disease cases. We used whole-exome data for two Mendelian disorders for which the causal gene is known. Considering only genes that contained variants in all cases, the VEST gene score ranked dihydroorotate dehydrogenase (DHODH) number 2 of 2253 genes in four cases of Miller syndrome, and myosin-3 (MYH3) number 2 of 2313 genes in three cases of Freeman Sheldon syndrome.

**Conclusions:**

Our results demonstrate the potential power gain of aggregating bioinformatics variant scores into gene-level scores and the general utility of bioinformatics in assisting the search for disease genes in large-scale exome sequencing studies. VEST is available as a stand-alone software package at http://wiki.chasmsoftware.org and is hosted by the CRAVAT web server at http://www.cravat.us

## Background

The identification of mutations and genes underlying human genetic disease continues to be a very active area of research. Advances in DNA sequencing technology have made it possible to rapidly identify all genetic variants in an individual exome. This large-scale enumeration of variants poses considerable difficulty for the identification of disease-causing variants, as they must be singled out from among a large pool of candidates.

Rare non-synonymous single nucleotide variants (NS-SNVs) that alter protein sequence are particularly strong candidates for disease-causing variants [1-3]. Experimental assessment of protein activity for mutated proteins is very difficult, and is further impeded by the large number of NS-SNVs revealed by exome sequencing studies; in general, sequencing identifies 3000 - 6000 NS-SNVs per exome. This has motivated the development of many statistical and computational methods for evaluating the functional impact of non-synonymous changes on proteins. The methods fall broadly into two categories, those that score mutations on the basis of biological principles (SIFT [4], MutationAssessor [5], MAPP [6], PANTHER [7], among others), and methods that use existing knowledge about the functional effects of mutations in the form a training set for supervised machine learning (PolyPhen2 [8], SNAP [9], SNPs3D [10], MutPred [11], MutationTaster [12], among others) [3]. These methods are known to perform well at distinguishing Mendelian disease mutations from common single nucleotide polypmorphisms [13] and usually offer either a numeric score that represents the predicted functional impact of an amino acid substitution, or a probability that the substitution is deleterious to the protein. Mutation scores can be used to substantially reduce the number of candidate disease-causing mutations detected in exome sequencing studies, but additional evidence is still needed to identify the causal mutation unequivocally. One recent strategy that has had considerable success in identifying both causal genes and mutations underlying Mendelian disorders is to assess several disease exomes together in order to reduce the number of candidate disease genes. Sequencing several exomes from unrelated individuals with the same Mendelian disorder can substantially reduce the list of candidate mutations/genes since all cases are expected to result from mutations affecting the same gene. Ng *et al*. sequenced the exomes of four individuals with Miller syndrome [14] and four individuals with Freeman Sheldon syndrome [15] to identify the subset of genes mutated in all individuals in each group. In both studies, mutations in the remaining genes were filtered against dbSNP and variants detected in 8 individuals from the HapMap project in order to eliminate neutral human variation. Using this strategy, Ng *et al*. were able to identify DHODH, encoding the enzyme dihydroorotate dehydrogenase, as the causal gene underlying the four Miller syndrome exomes and MYH3, encoding myosin-3, as the gene underlying the four Freeman Sheldon syndrome exomes. MYH3 had previously been identified as the gene underlying Freeman Sheldon syndrome via careful selection of candidate causal genes for sequencing based on similarity to other Mendelian disorders with known genetic causes [16]. The work by Ng *et al*. demonstrated that a data-driven approach can be useful in identifying disease genes when there is no a priori list of candidate genes.

We postulated that it should be possible to identify causal genes on the basis of enrichment for functional mutations across disease exomes without filtering out common variants first. Our reasoning was that enrichment for functional mutations in a gene underlying a common phenotype would provide a stronger signal than functional mutations occurring in genes unrelated to the phenotype. To test this hypothesis, we applied our supervised machine learning-based method for predicting functional mutations to the variants observed in the four Miller syndrome exomes and three of the four Freeman Sheldon syndrome exomes described by Ng *et al*. Our Variant Effect Scoring Tool (VEST) was trained on a positive class of missense variants from the Human Gene Mutation Database [17] (2012v2) and a negative class of common missense variants detected in the Exome Sequencing Project population [18]. VEST performed well at identifying functional mutations in careful benchmark experiments, with an area under the Receiver Operating Characteristic of 0.92. One difference between VEST and other existing methods for predicting functional mutations is that VEST uses a statistical hypothesis testing framework to assign p-values to predictions. In order to identify the causal genes, we aggregated VEST p-values for all mutations by gene across all disease exomes to create a gene-level statistic. Ranking genes based on this statistic placed both MYH3 and DHODH among the top 2 candidate causal genes.

Our gene prioritization approach differs from current statistical methods for gene burden testing (reviewed in [19,20]) in that we do not require a control population to prioritize genes, although a matched control population, if available, is useful for eliminating genes that are artifactually significant as a result of sequencing and variant calling errors. In addition, we show that the causal genes can be identified without including allele frequency information, although we expect that using allele frequency information to filter the list of variants detected in exome sequencing should make it easier to detect disease genes.

The effectiveness of gene burden algorithms is often evaluated on the basis of statistical power to detect simulated disease genes [21]. We evaluated our gene score using this approach by simulating disease genes over a range of parameters representative of real exome sequencing data. We also performed simulations to evaluate the potential consequences of VEST misclassification error for correct disease gene prioritization. The results of the simulations support the potential of our p-value-based gene score to correctly identify Mendelian disease genes using VEST functional predictions.

## Results and discussion

### VEST

The Variant Effect Scoring Tool (VEST) is a new method for prioritizing missense mutations that alter protein activity. VEST uses a supervised machine learning algorithm, Random Forest [22,23], to identify likely functional missense mutations. The training set is a positive class of missense variants from the Human Gene Mutation Database and a negative class of common missense variants detected in the Exome Sequencing Project (ESP) population. An allele frequency of 1% or higher is considered common for this work. All mutations are described by a set of 86 quantitative features.

Although the VEST training set is designed to identify missense mutations that alter protein activity, its construction includes the additional context of selective difference between disease and common variants. The disease class from HGMD is enriched for variants under purifying selection, whereas the neutral class from ESP common variants includes variants under positive selection in the human population in addition to truly neutral variants [24]. If we assume that the VEST classifier can generalize to mutations not in its training set, it should be able to take a set of variants from a sequenced exome and prioritize functional mutations which are similar to disease mutations over those which are similar to neutral mutations or to functional mutations under positive selection.

#### VEST performance

**Figure 1:**
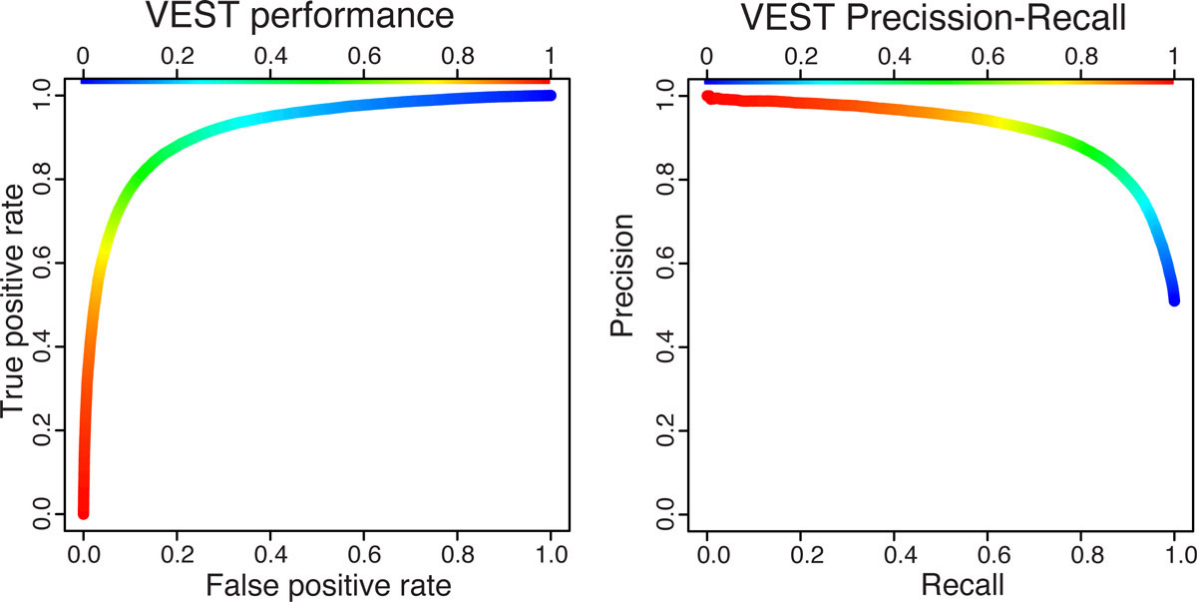
**VEST Classifier performance**. Receiver Operating Characteristic (left) and precision-recall curve (right) for VEST were constructed using 5-fold gene holdout cross validation on the VEST training set. The AUC statistics for these two curves were both 0.92 indicating that the VEST classifier has good sensitivity and specificity for identifying mutations with functional consequences for protein activity.

In our experiments, VEST is able to generalize well, when applied to mutations outside of its training set. We estimate VEST generalization error stringently, using gene-holdout cross-validation, similar to Capriotti et al. [25]. We believe that gene-holdout cross-validation helps control bias in generalization error estimates. Although the features used to describe each mutation do not explicitly contain gene level features, we expect to see greater correlation between feature vectors for mutations in the same gene relative to feature vectors for mutations in different genes. Thus, by placing all mutations occurring in the same gene into the same cross-validation partition, we prevent the possibility of overestimating VEST performance based on the elevated correlation between mutations in the same gene. We quantify VEST's performance with a Receiver Operator Characteristic (ROC) [26] and a Precision-Recall (PR) curve [27] (Figure 1). The area-under-the-curve (AUC) statistic for these curves provides a simple measure to summarize classifier performance. VEST achieves an ROC AUC of 0.92, and at a VEST score of 0.5 over 80% of the disease class mutations are correctly classified whereas fewer than 20% of the neutral class mutations are misclassified as functional.

We compared VEST to two popular methods for predicting functional mutations, SIFT 4.0 [4] and PolyPhen2 [8] using a simple holdout strategy to achieve an unbiased comparison. The holdout strategy is necessary to ensure that trained classifiers do not have an unfair advantage in predicting benchmark set mutation class membership as can occur when benchmark mutations are included in the classifier training set. PolyPhen2 uses supervised machine learning to train a classifier, while SIFT4.0 does not. It was therefore necessary to design a benchmark set that did not include any mutations in the PolyPhen training set. Once again, we assumed that a higher correlation between the feature vectors of mutations in the same gene could lead to an advantage for correctly predicting mutation class, so we constructed our benchmark set such that only mutations in genes that had no mutations in the PolyPhen2 classifier training set were selected for the benchmark set.

**Figure 2:**
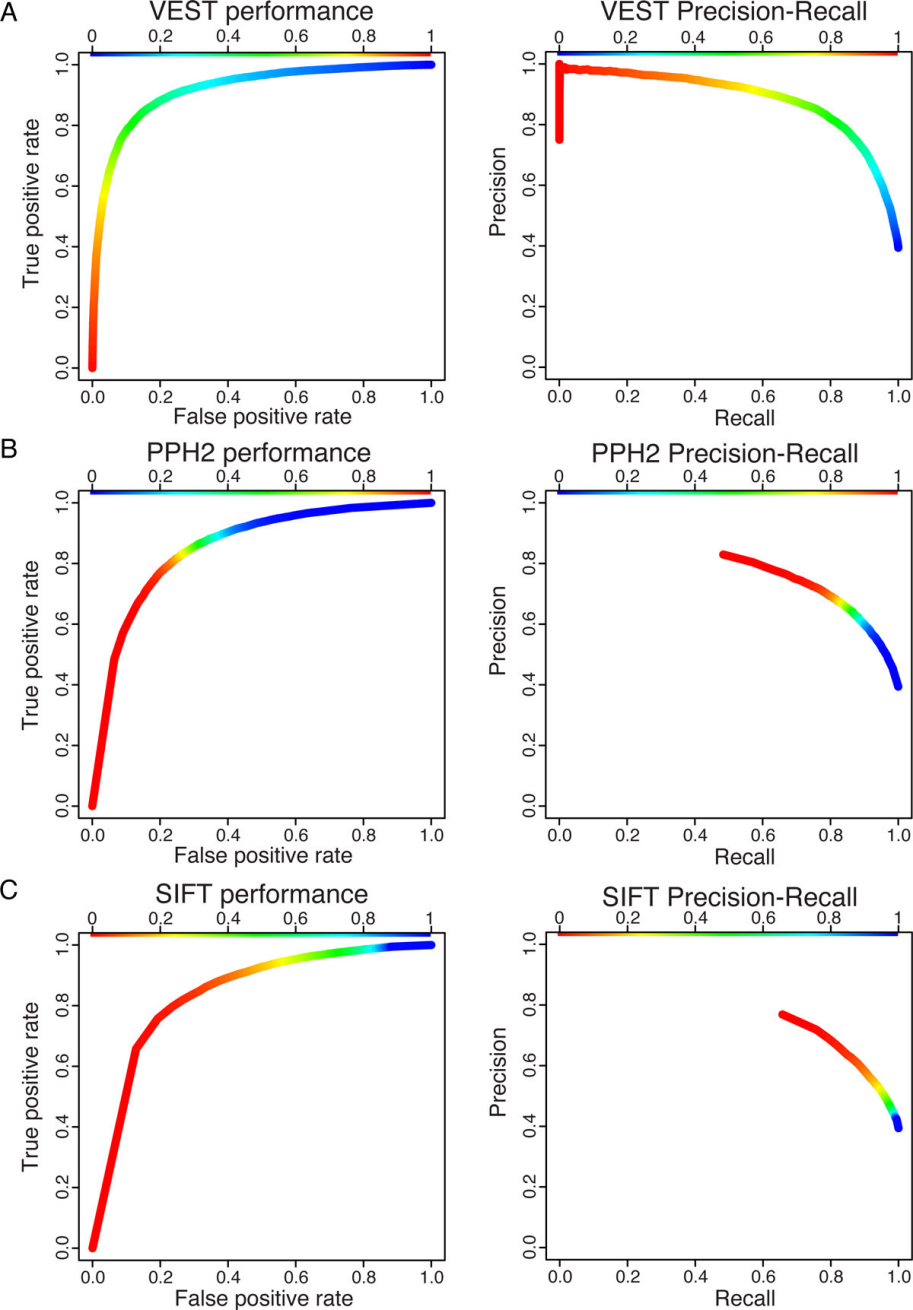
**Comparison of VEST with popular methods PolyPhen2 and SIFT4.0**. Receiver Operating Characteristic (left) and precision-recall curve (right) for VEST (A), PolyPhen2 (B) and SIFT4.0 (C). The color bar for SIFT is reversed since a low SIFT score corresponds to positive class prediction. ROC AUC is 0.92, 0.85, 0.84 for VEST, PolyPhen2 and SIFT respectively. PR AUC is 0.88, 0.76, 0.72 for VEST, PolyPhen2 and SIFT respectively.

PolyPhen2 provides two trained classifiers, HumDiv and HumVar, for evaluating missense mutations. HumDiv is recommended for assessing rare alleles detected in exome sequencing, while HumVar is recommended for identifying variants underlying Mendelian disorders. Therefore, in order to compare VEST to PolyPhen2, we created two benchmark datasets, one to be used for comparison to the HumDiv classifier and the other for comparison to the HumVar classifier (Table 1). To ensure that VEST did not have an unfair advantage, the VEST classifiers used for benchmarking were then trained only on the mutations from the training set remaining after benchmark set construction. All classifiers (SIFT, PolyPhen2 and VEST) were used to score the benchmark set mutations. Both PolyPhen2.0 and SIFT4.0 were unable to score a subset of the benchmark mutations (Table 2), so a direct comparison of classifier performance was only possible on the subset of benchmark mutations scored by all three methods. Results of the benchmark comparison are shown in Figure 2 and described in Table 3.

**Table 1:** Dataset composition.

Class	Training set	Benchmark test set	Benchmark training set
Disease	47724	32841	14883
Neutral	45818	31018	14800

The VEST training set comprises a balanced number of disease-causing and neutral missense mutations. In order to compare VEST performance to other methods, the VEST training set was divided into a benchmark test set for evaluating methods, and a benchmark training set, used for training a VEST classifier for unbiased comparison.

**Table 2:** Benchmark mutation coverage.

	Benchmark	
**Method**	**Disease**	**Neutral**

VEST	100%	100%
PolyPhen2	89%	88%
SIFT4.0	57%	94%
All 3	51%	84%

Percentage of the benchmark test set mutations that could be scored by each of the methods compared.

**Table 3:** Classifier performance. Classifier performance on the benchmark test set. Area under the curve (AUC) statistics for Receiver Operating Characteristics and Precision-Recall curves are shown

	Performance on benchmark		Performance on benchmark (subset scored by all methods)	
**Method**	**ROC AUC**	**PR AUC**	**ROC AUC**	**PR AUC**

VEST	0.912	0.919	0.917	0.884
PolyPhen2	0.857	0.840	0.854	0.762
SIFT4.0	0.835	0.715	0.838	0.724

#### Predicting functional mutations with VEST

VEST assigns variants a score between 0 and 1, where 1 indicates a confident prediction of a functional mutation. We use a statistical hypothesis testing framework to assess the significance of each score and adjust for multiple testing as described previously [28]. Briefly, a set of missense mutations representative of neutral human variation provide a null model for testing the null hypothesis that a given VEST score indicates a neutral mutation. If the VEST score falls into the right tail (since large VEST scores indicate a prediction of disease) we can uses the null model to assess the probability of a neutral mutation from our null model receiving a VEST score of the same magnitude by chance. In general we expect to make a large number of comparisons to the null distribution because the number of missense mutations detected in exome sequencing is large, therefore we estimate a false discovery rate (FDR) cutoff for each p-vale using the Benjamini-Hochberg method [29]. The p-value and FDR associated with each mutation can be used to determine a score cutoff for accepting a functional prediction. The precise cutoff is subjective, allowing the user to determine an acceptable tradeoff between the number of candidate functional mutations identified, and the estimated rate of false positive predictions. For this work, we modeled neutral missense mutations using common variants (AF ≥ 1%) observed in the 1000 Genomes Project [30], and used the VEST score distribution for these mutations to estimate p-values.

#### Feature selection

We attempted to improve VEST classifier performance using feature selection to remove features that were uninformative for discriminating between disease and neutral mutation classes. The predictive power of individual features was assessed using mutual information (MI), which provides a measure of the amount by which the knowledge provided by the feature decreases the uncertainty about the mutation class label [31]. To acquire an unbiased estimate of improved performance, we partitioned the training set into 3 parts: one for estimating mutual information for the 86 features, one for training a classifier with selected featuers, and one for evaluating the resulting change in classifier performance. We selected 47 features based on a threshold of MI ≥ 0.001 bits. Using only the 47 selected features for classifier training gave a slight boost to sensitivity, but reduced classifier specificity (ROC AUC = 0.94 PR AUC = 0.89). MI thresholds of MI ≥ 0 bits and MI ≥ 0.01 bits were also tested (ROC AUC = 0.81 PR AUC = 0.72 and ROC AUC = 0.94 PR AUC = 0.88 respectively). Based on these findings, we elected to use the full set of 86 features for VEST classifier construction.

### Gene prioritization in Mendelian Disorders

#### Mendelian exome processing

We acquired raw DNA sequence reads for seven Mendelian disease exomes from dbGaP [32], four Miller syndrome exomes and three Freeman-Sheldon syndrome exomes. Read mapping and variant calling were performed as described in Methods. Results of variant calling are described in Table 4. The numbers of variants and mutated genes we found in these exomes are slightly smaller than the numbers reported in the original publications. This difference was due to the variant calling pipeline we used, which differs from that used in the original publications. In addition, we used a phred-quality cutoff ≥ 30, similar to that used in the Freeman Sheldon syndrome study, whereas the original Miller syndrome study used a cutoff ≥ 20.

**Table 4:** Variant calling results.

Exome	SNV calls	cSNVs	InDel calls	Missense	Truncating	Mutated genes	Common mutated genes
Miller Syndrome 1	18256	14744	391	6569	98	4213	2258
Miller Syndrome 2	17403	14438	381	6409	113	4096	2258
Miller Syndrome 3	18367	14907	428	6575	106	4210	2258
Miller Syndrome 4	17074	14166	373	6377	111	4059	2258
Freeman Sheldon 1	19957	15383	567	6891	139	4310	2314
Freeman Sheldon 2	18929	14730	502	6618	123	4151	2314
Freeman Sheldon 3	15125	12361	371	5485	112	3675	2314

Summary of variant calling results for four Miller syndrome exomes and three Freeman Sheldon syndrome exomes.

#### Mendelian exome variant scoring

We mapped variants detected in the four Miller syndrome exomes and the three Freeman Sheldon syndrome exomes onto proteins. Variants that resulted in an amino acid substitution were grouped into missense mutations and truncating mutations. For this work, four types of mutation were considered truncating: single nucleotide changes resulting in nonsense and nonstop mutations, frameshift mutations, and deletions eliminating a splice site. Variants resulting in silent changes or in-frame insertions and deletions were discarded. Missense mutations detected in each exome were scored with VEST. Truncating mutations were not initially considered since VEST is designed specifically for missense mutations. For the purpose of identifying DHODH and MYH3 from the missense mutations detected in the disease exomes, we trained two custom VEST classifiers. The first classifier used a training set filtered to eliminate all MYH3 mutations; and the second was filtered to eliminate all DHODH mutations. Using these classifiers to score missense mutations ensured that DHODH and MYH3 mutations did not receive artificially significant VEST scores because of overlap with the training set. This was necessary to ensure an unbiased assessment of our gene score for identifying candidate causal genes.

#### Selecting a method to aggregate VEST scores by gene

We tested three methods for aggregating VEST mutation scores into gene scores: the average VEST score, Fisher's method [33] and Stouffer's Z-score [34]. We compared each method on the basis of gene rank of the causal genes underlying the four Miller exomes (DHODH) and the three Freeman Sheldon exomes (MYH3). We found that Stouffer's Z most consistently ranked the causal genes highest. Fisher's method aggregates the log2 of the p-value of each mutation and hence low p-values make a larger contribution to the final score than higher p-values. As a consequence, genes with a large number of mutations of which only a small number receive functional VEST scores can still receive small p-values, leading to a larger list of candidate disease genes. Stouffer's method appears to provide the best gene prioritization for Mendelian disease genes. Both methods for aggregating p-values outperformed the average VEST score approach (Table 5).

**Table 5:** Gene rank and p-value assigned to causal gene in two Mendelian disorders.

Disorder/Gene	Mean VEST Rank	Mean VEST score	Fisher Rank	Fisher p-value	Stouffer Rank	Stouffer p-value
*All genes *						
Miller syndrome/DHODH	112	0.71	7	11.6e-07	3	1.9e-06
Freeman Sheldon syndrome/MYH3	124	0.67	3	3.7e-14	3	9.3e-07
*Dominant model *						
Miller syndrome/DHODH	15	0.71	5	4.4e-08	2	1.9e-06
Freeman Sheldon syndrome/MYH3	12	0.67	3	3.7e-14	2	9.3e-07
*Recessive model *						
Miller syndrome/DHODH	12	0.71	4	9.8e-06	2	1.9e-06
*Dominant model including truncating *						
Miller syndrome/DHODH	11	0.75	10	7.0e-11	3	2.5e-08
Freeman Sheldon syndrome/MYH3	19	0.67	10	3.8e-14	12	9.3e-07
*Recessive model including truncating *						
Miller syndrome/DHODH	9	0.75	10	7.14e-10	3	2.5e-08

Ranks and p-values for the genes underlying Miller syndrome and Freeman Sheldon syndrome for three gene lists: all genes harboring missense mutations, genes with a missense mutation in at least one copy in all disease exomes (dominant model), and genes with a missense mutation in both copies in all disease exomes (recessive model). Genes were ranked using two scoring methods, Fisher's method and Stouffer's Z score.

#### Accounting for Mendelian disease models

**Figure 3:**
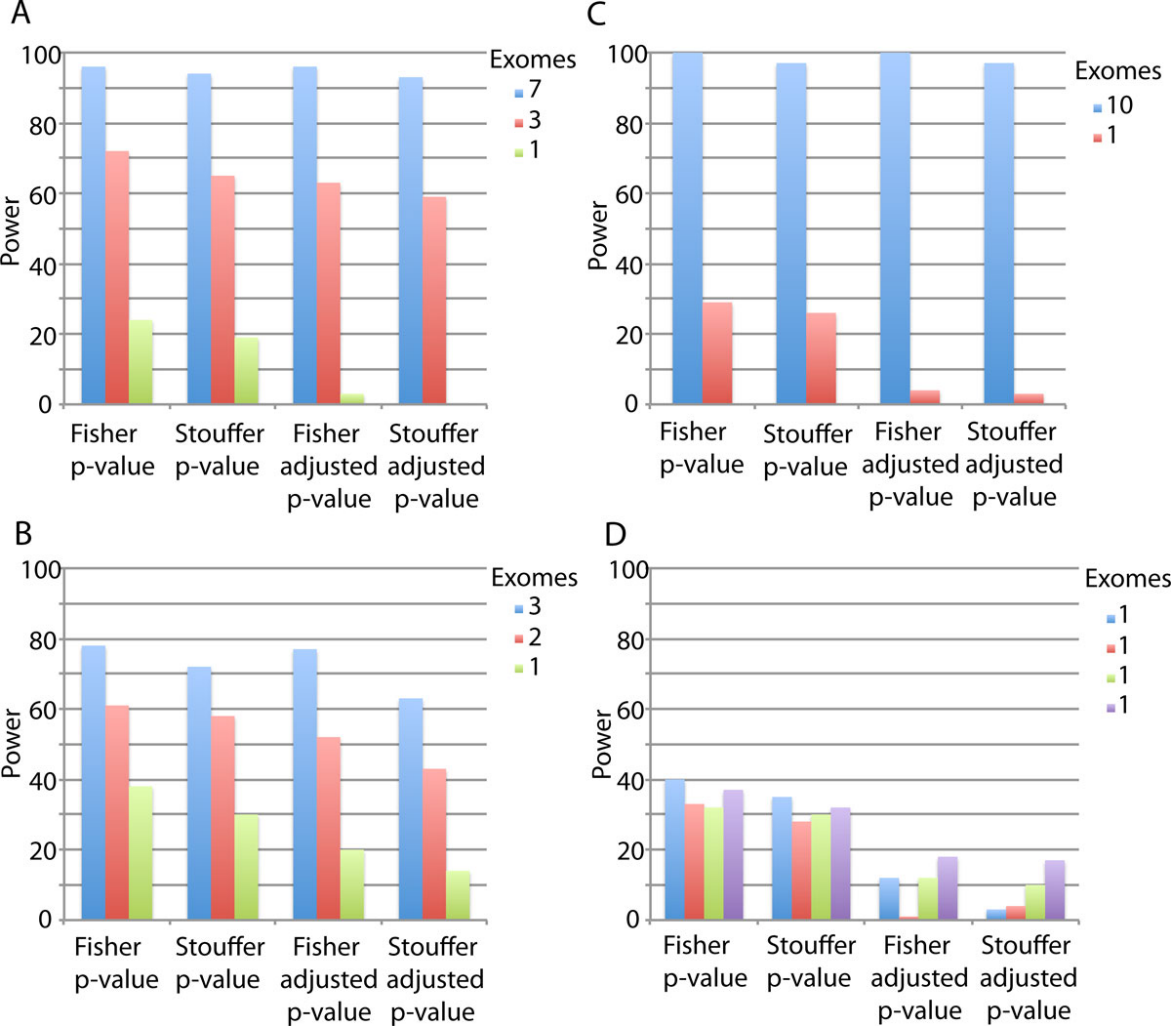
**Power to detect disease genes in simulated cases of locus heterogeneity**. Estimated power to detect disease genes in the presence of locus heterogeneity when A) seven, three and one exomes share disease genes B) three, two and one exomes share disease genes C) ten and one exomes share disease genes D) each of four exomes results from a distinct disease gene. In each case gene p-values acquired using both Fisher's and Stouffer's methods are compared. Power is shown for raw p-values as well as Benjamini-Hochberg adjusted p-values. The height of each bar corresponds to the number of simulations in which the gene received a p-value or adjusted p-value *<*0.05.

We first attempted to identify DHODH and MYH3 by simply considering all mutations detected across the disease exomes. Since both Miller syndrome and Freeman Sheldon syndrome are Mendelian disorders, we also tried pre-filtering the list of mutated genes using possible disease models. For this work, we considered autosomal dominant and autosomal recessive models. Patterns of Mendelian disease segregation in families can provide clues as to the appropriate disease model. In the absence of this information, a simple autosomal dominant model represents a conservative choice since all genes harboring at least a single mutation in all disease exomes must be considered. Since Miller syndrome had previously been observed to have an autosomal recessive pattern of inheritance, we also tried a recessive model where only genes harboring at least two mutations in each exome were considered. Using a disease model to filter the list of candidate genes improved the ranks of each mutation (Table 5), but the recessive model, while reducing the number of genes taken into consideration from 2258 to 1267, did not improve our ability to detect DHODH. Some Mendelian disorders display locus heterogeneity, where the same disorder in two or more individuals results from mutations in distinct genes. We first assessed the potential of our gene score to identify multiple disease genes in a setting of locus heterogeneity by combining the three Freeman Sheldon syndrome exomes with the four Miller syndrome exomes prior to ranking mutated genes. When all seven exomes were considered simultaneously and any gene mutated in at least two individuals was scored, we found that MYH3 ranked second and DHODH ranked fourth out of 5561 candidate genes. Next we tried combining one Freeman Sheldon syndrome exome with all four Miller syndrome exomes, and vice versa. In these cases, the disease gene in the majority of exomes ranked in the top 3 genes while the disease gene from the single exome ranked anywhere from 26 to 710 (out of ~6400 genes) depending on the magnitude of the VEST score for the disease mutation (Table 6). Finally, we performed simulations to estimate the power of our approach to identify disease genes at different degrees of locus heterogeneity. We find that locus heterogeneity does reduce power to detect disease genes that are mutated in only a small fraction of disease exomes (Figure 3). In our simulations, disease genes p-values are smallest when the Z-score sampled for the disease mutation is large, and when the number of non-disease mutations observed in the disease gene is low, suggesting that disease genes that are not frequently mutated and harbor disease mutations of strong effect will be more easily detected.

**Table 6:** Mendelian disease gene detection under locus heterogeneity.

MYH3 mutation VEST scores	Fisher Rank (MYH3)	Stouffer Rank (MYH3)	Fisher Rank (DHODH)	Stouffer Rank (DHODH)
0.045, 0.993	5	6	8	3
0.045, 0.963	144	710	7	3
0.045, 0.963	142	695	7	3

**DHODH mutation VEST scores**	**Fisher Rank (MYH3)**	**Stouffer Rank (MYH3)**	**Fisher Rank (DHODH)**	**Stouffer Rank** **(DHODH)**

0.33, 0.98, 0.859	26	27	3	3
0.33, 0.952, 0.523	53	118	3	3
0.33, 0.98, 0.859	26	27	4	3
0.33, 0.636	582	554	3	3

Gene ranks for DHODH and MYH3 using the Fisher and Stouffer gene score methods when Freeman Sheldon syndrome exomes and Miller syndrome exomes are combined as one versus all. The top half of the table shows the results of combining individual Freeman Sheldon exomes with all four Miller syndrome exomes, while the bottom half shows the results of combining each Miller syndrome exome with all three Freeman Sheldon syndrome exomes. The first column reports VEST scores for missense mutations in the causal gene in the single exome.

#### Including truncating mutations

The VEST classifier is specifically designed to prioritize missense mutations. However, truncating mutations also alter protein activity and contribute to human genetic disease. Each truncating mutation *t *was assigned a score

(1) [1-AF(t)]*max(M),

where *AF *is allele frequency and **M **represents the VEST scores of all missense mutations observed in the sequenced exomes. Thus, low frequency truncating events received high VEST scores. We then estimated p-values for the truncating mutation scores using the 1000 Genomes null model. We then recalculated gene scores using p-values for both missense and truncating mutations. When truncating mutations were included, DHODH and MYH3 dropped in rank among prioritized genes to 3 and 12 respectively (Table 5). This is not surprising, since a number of the truncating mutations are present at low frequency or result from what appear to be private mutations and therefore receive VEST scores close to 1. It should be noted that indel calling is difficult [35] and indel calling algorithms are thought to have a high false positive rate for calling frameshift events [36]. The possibility that novel frameshift mutations detected by variant calling pipelines may be enriched for false mutations suggests that it will be important to develop a better scoring algorithm for truncating mutations when including them for gene prioritization.

#### Effect of null distribution on gene score

**Figure 4:**
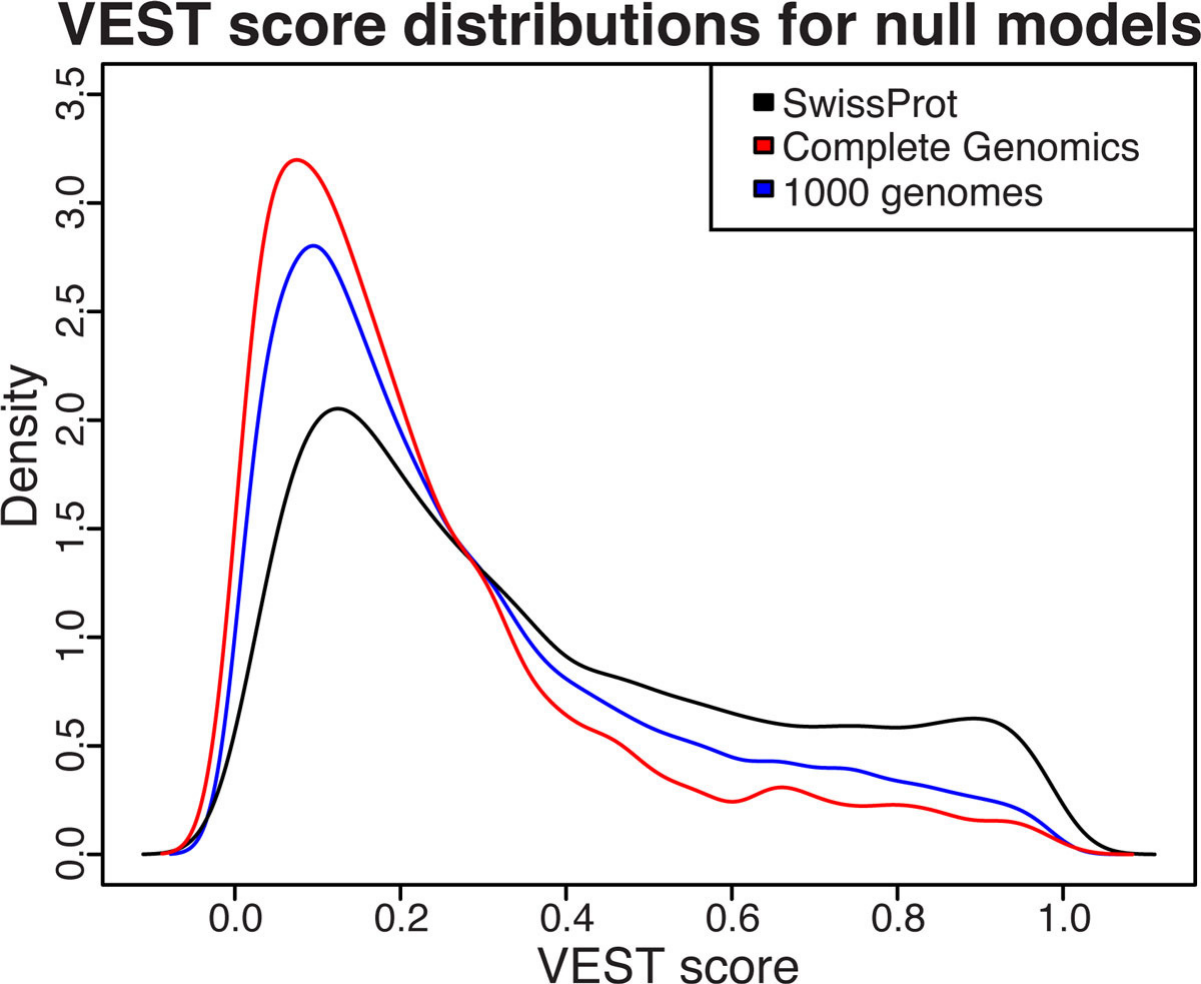
**Comparison of VEST score distribution for three empirical null models**. Density plots created from VEST score distributions for three empirical null models representing neutral human missense variation. Null model mutations were filtered to remove overlap with the VEST training set, then scored with the VEST classifier. The Swissprot-based null shows an enrichment for large VEST scores in the right tail, indicating predicted functional mutations.

The choice of null model for assessing VEST score significance can directly influence the gene score. This is because the gene score is estimated from p-values, and the magnitude and dispersion of the p-values for a set of VEST scores depend on the shape of the null distribution used to assess score significance. VEST score distributions for three different empirical null models are shown in Figure 4: one generated from common variants observed in the 1000 Genomes Project, one comprising neutral polymorphisms from the SwissProt variant pages [37], and another consisting of variants from the Complete Genomics 69 exome diversity panel [38]. We find that using different null models to estimate VEST score p-values does influence the ranking of MYH3 and DHODH among the candidate disease genes (Table 7). The 1000 Genomes Project null model looks similar to the Complete Genomics null model even though the two datasets were sequenced at very different coverage (a range of 2X to 4X for 1000 Genomes versus a range of 51X to 89X for Complete Genomics). This suggests that coverage depth is unlikely to have introduced systematic bias into the null model when only variants at allele frequencies ≥ 1% are considered.

**Table 7:** MYH3 and DHODH rankings using different null models.

Gene	FisherRank	FisherP	FisherBHFDR	StoufferRank	StoufferP	StoufferBHFDR
*Swissprot null model*						
DHODH	4	3.8e-05	0.05	2	0.001	1.00
MYH3	5	1.7e-05	0.05	4	0.021	1.00
*1000 genomes null model*						
DHODH	5	4.4e-08	0.05	2	1.9e-06	0.05
MYH3	3	3.8e-14	0.05	2	9.3e-07	0.05
*Complete genomics null model*						
DHODH	5	4.6e-08	0.05	2	1.9e-06	0.05
MYH3	15	2.0e-06	0.05	5	2.4e-03	1.00

Ranks of MYH3 and DHODH differ slightly when different null models are used to estimate p-values for VEST mutation scores.

Interestingly, the null model based on SwissProt polymorphisms showed more enrichment for functional VEST scores than the other null models. This suggests that some SwissProt polymorphisms may not be functionally neutral, or that VEST misclassifies a subset of them because of some difference between the SwissProt polymorphisms and the common variants used to train the VEST classifier.

#### Assessing the power of the gene score to detect disease genes

**Figure 5:**
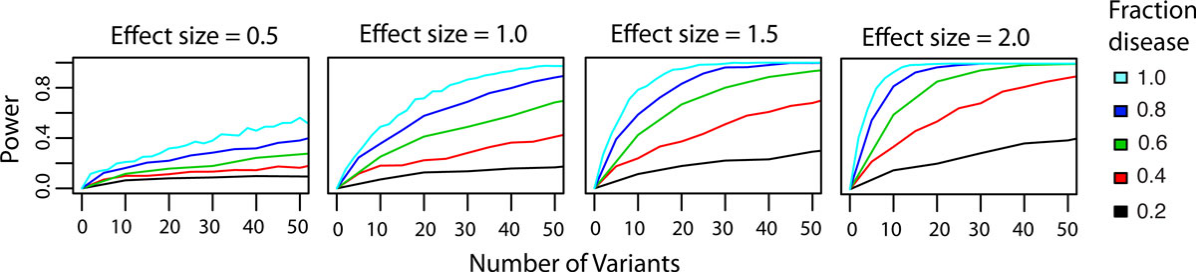
**Sensitivity of gene score to mutation count and fraction of functional mutations at different effect sizes**. Power to detect disease genes was estimated using simulations in R. Mutation counts and fraction of functional mutations were varied at four different effect sizes (0.5, 1.0, 1.5 and 2.0). A distinct plot represents the results of the simulation for each effect size. The legend on the top right shows the fraction of disease mutations simulated in each gene.

We attempted to quantify the power of our gene score for identifying disease genes using simulations implemented in R [39]. For our simulations, we represent each mutation by a Z-score. Neutral mutation Z-scores are sampled from the standard normal distribution, while disease mutation Z-scores are sampled from a standard normal distribution with a mean shift. We simulate a population of 1000 disease genes by sampling mutations from both distributions and then calculating a gene level p-value using Stouffer's Z-score or Fisher's method. In order to use Fisher's method, Z-scores are first converted to p-values. We estimate the power of each gene score as the fraction of simulated disease genes receiving a p-value *<*0.05. We assessed the sensitivity of the two gene scoring approaches to three parameters of the simulation: the mean shift between the null and disease Z-score distributions (the effect size), the total number of mutations per gene, and the fraction of the mutations that are sampled from the disease Z-score distribution. The mean shift between null and Z-score distributions is a proxy for the difference in the mean VEST scores for neutral mutations versus functional mutations. The effect of varying these parameters on power to identify disease genes is shown in Figure 5. Power is plotted on the y-axis, and the total number of mutations per gene is plotted on the x-axis. Each plot represents a different effect size (0.5,1.0,1.5,2.0 from left to right). The family of curves in each plot represents a population of genes simulated with a different fraction of mutations sampled from the disease Z-score distribution. Z-score distributions generated from the unbiased gene-holdout cross-validation VEST scores for the training set suggest that we can expect an effect size greater than 1.7 for Mendelian disease mutations.

**Figure 6:**
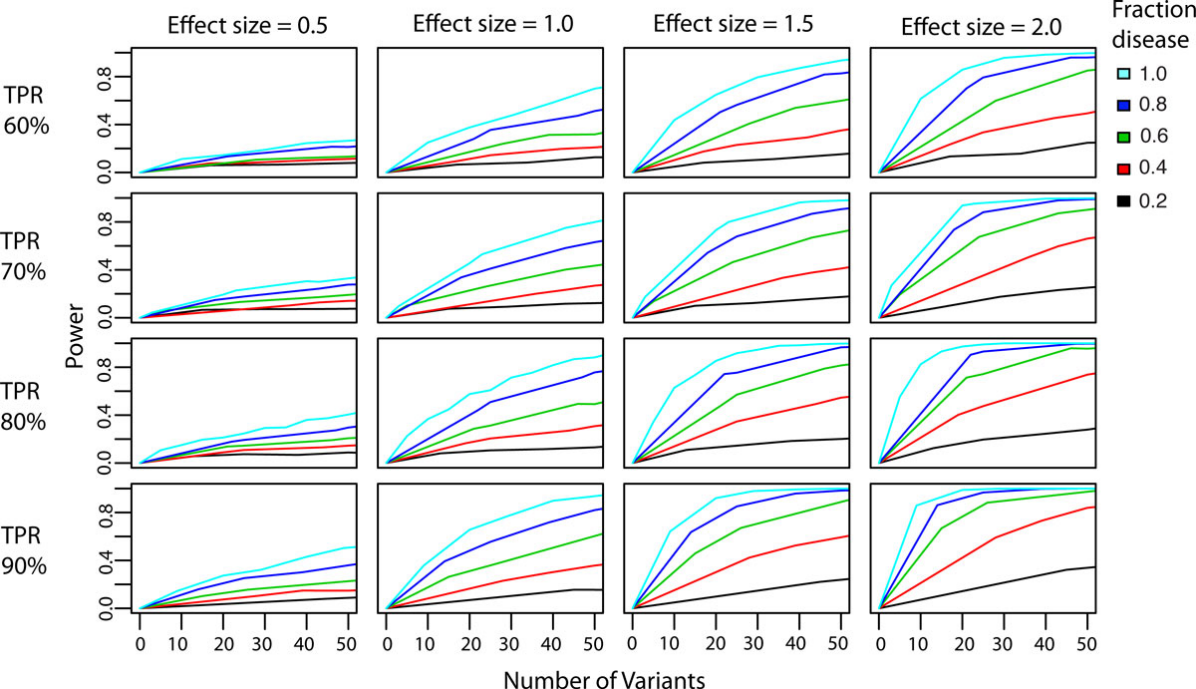
**Sensitivity of gene score to VEST classification error**. Power simulations were repeated with an additional parameter: VEST true positive rate (TPR). Four TPRs were selected based on VEST generalization error estimates. A set of simulation is shown for each of the four points (60%, 70%, 80% and 90%). As expected, power to detect disease genes decreases as the TPR decreases.

Disease gene identification is clearly sensitive to the effect size of disease mutations. The proportion of mutations observed in a gene that are disease related is also a key determinant of power, therefore it may be easier to detect disease genes if mutations are pre-filtered to remove common mutations unlikely to contribute to disease. Power increases as the number of mutations increases, which may indicate that increasing the number of disease exomes will result in improved ability to identify causal genes. We also assessed the effect of VEST misclassification error on the power to identify disease genes. We selected four points from the ROC curve constructed to estimate VEST generalization error. Each point represents a reasonable tradeoff between classifier sensitivity and specificity (TPR = 60%, FPR = 5%; TPR = 70%, FPR = 10%; TPR = 80%, FPR = 15%; TPR = 90%, FPR = 25% where TPR=True Positive Rate and FPR = False Positive Rate). We repeated our power simulation at each of these misclassification rates. The number of disease mutations sampled for each gene was multiplied by true positive rates of 90%, 80%, 70% and 60% to simulate the effects of misclassification. The results of these simulations are shown in Figure 6. Each row of plots represents the power to detect disease genes at different VEST true positive rates. Misclassification error does reduce the power to detect disease genes, but only marginally. The largest decreases in power occur at a true positive rate of 60% and are on the order of 20%. In general, if both the effect size and the fraction of disease mutations are small, disease genes will be difficult to detect regardless of misclassification. If effect size and fraction of disease mutations are large, the effects of misclassification are negligible.

#### Contrasting gene scores in Freeman Sheldon syndrome and Miller syndrome

Mutations detected in exome sequencing include artifactual mutations resulting from DNA sequencing and variant calling errors. These false variants can confound the search for true disease causing mutations/genes. Since sequencing errors are not associated with disease phenotype, it is often possible to filter them out by comparing to a control population. Specifically, systematic sequencing error should be apparent because it will result in similar rates of false mutations in both disease exomes and controls, while the true causal mutations should be present in only the disease exomes. To effectively use this approach, both disease exomes and control exomes must be processed using the same DNA sequencing technologies and variant calling pipelines [40].

Since both the Freeman Sheldon syndrome exomes and the Miller syndrome exomes were sequenced using the same technology, we hypothesized that genes not causally related to these phenotypes might have received similar gene scores in both groups. If so, this could enable us to remove from consideration any genes that received significant scores due to false mutations resulting from DNA sequencing and variant calling errors. In order to prioritize genes that behave differently in the two groups of exomes, we fit a linear model using gene score p-values. When genes are required to meet the criteria for a dominant disorder (at least one mutation per exome), only 1640 genes were common to the two groups, including MYH3 but not DHODH. MYH3 received the largest negative residual with the linear model, identifying it as being enriched for functional mutations in Freeman Sheldon syndrome relative to Miller syndrome (Table 8). Furthermore, many of the top scoring genes that were initially prioritized due to false mutations, including CDC27, CTBP1, and OR4C3 [41-43] receive residuals close to 0 indicating that they harbor a similar profile of mutations in both exome sets and are therefore unlikely to be causally related to either disorder.

**Table 8:** Top genes in Freeman Sheldon syndrome exomes after comparison to Miller syndrome Exomes.

Rank	GeneSymbol	Residuals	FSS p-value	Miller p-value
1	MYH3	-0.92	0.000	0.946
2	MYH7B	-0.72	0.032	0.745
3	GCN1L1	-0.28	0.049	0.247
4	MLL3	-0.23	0.032	0.164
5	POTED	-0.22	0.031	0.159
6	FRG2C	-0.20	0.013	0.108
7	KCNJ18	-0.17	0.001	0.058
8	SLC12A3	-0.13	0.044	0.061
9	BCLAF1	-0.12	0.005	0.008
10	OR4C3	-0.12	0.002	0.004

After comparing gene scores in Freeman Sheldon syndrome (FSS) exomes to Miller syndrome exomes using a linear model, genes are re-sorted on residuals. Here, small negative residuals indicate genes that were more significant in Freeman Sheldon syndrome exomes relative to Miller syndrome exomes.

## Conclusions

Whole exome sequencing studies for the characterization of disease variation uncover thousands of candidate causal mutations per individual. In this work, we have developed a new, accessible tool, VEST, for prioritizing functional mutations among those detected through exome sequencing. We show that simple aggregation of VEST prediction p-values across genes and disease exomes can be used to identify disease genes in Mendelian disorders. In two Mendelian disorders with known causation, our gene score places the causal genes among the top 5 candidate genes with no prior filtering to reduce the list of candidates, and in the top 2 when only genes that meet Mendelian criteria are considered. This serves as proof of principle that bioinformatics methods for scoring mutations can be used directly for disease gene discovery in exome sequencing studies, through a simple application that could easily be automated. Disease related mutations and genes are more difficult to identify in complex disorders because there is less concentration of causal variants in individual genes, disease causing variants often have variable penetrance and environmental factors can greatly modify disease risk. The approach described here may nonetheless help to identify genes, or pathways consisting of multiple genes, that are enriched for functional mutations among individuals with complex disorders. Our method is distinct from existing methods that rely on differing allele frequencies in cases versus controls to identify causal genes. Allele frequencies are not required in our approach, but can be used to filter variants prior to VEST scoring and p-value aggregation. In addition, while matched controls are useful for removing genes that are functionally impaired but not associated with the specific phenotype, our approach is still useful when controls are not available. Further experiments are planned to determine the power of our approach for identifying genes involved in complex disease.

## Methods

### Read mapping and variant calling

Four Miller syndrome exomes [14] and three Freeman-Sheldon syndrome exomes [15] were acquired in the form of short read archive (SRA) files from dbGaP [32] (Miller syndrome study accession: phs000244.v1.p1 Freeman Sheldon syndrome study accesion: phs000204.v1.p1) and converted to fastq format using fastq-dump version 2.1.12 from the SRA Toolkit http://www.ncbi.nlm.nih.gov/Traces/sra/sra.cgi?cmd=show&f=software&m=software&s=software. The Miller syndrome and Freeman-Sheldon syndrome 76bp single end Illumina Genome Analyzer reads were pre-processed using the fastx toolkit [44] to only include reads with a Phred-like consensus quality 20 for 50 percent of the bases. The reads were then mapped to the reference genome (UCSC hg19) using the Burrows-Wheeler Aligner (BWA v0.6.1) [45]. The resulting outputs were sorted and indexed using samtools [46]. Unmapped reads and duplicates were then removed using the Bamtools [47] and picard software http://picard.sourceforge.net. The aligned positions with a Phred-like consensus quality value 30 were retained for downstream variant calling. The Genome Analysis Toolkit (v1.6-11-g3b2fab9) then realigned intervals around potential indels and re-calibrated the base quality scores [48,49]. GATK Analysis was limited to the human exome from the list of RefSeq exons plus an additional 10 bp at each splice site. GATK's Unified Genotyper module performed variant calling resulting in variant call format (VCF) files. The resulting VCF files (v4.1) were filtered through GATK's variant filter module using hard filter settings. SNP filtering removed calls based of the following criteria: (i) *MQ*0 *≥ *4&&((*MQ*0 = (1.0 * *DP*)) *>*0.1); (ii) *QUAL <*30.0 || *QD <*5.0 || *H Run >*5 || *SB >*-0.10); and (iii) cluster window size 10. Phasing for the SNP calls was determined using the GATK ReadBackPhasing algorithm restricted to the exon ranges at a phasing quality threshold of 20.0. Indel filtering removed calls based on the following criteria (i) *MQ*0 *≥ *4&&((*MQ*0/(1.0 * *DP*)) *>*0.1); (ii) *SB *≥ -1.0; (iii) *QUAL <*10.

### Variant mapping

Single nucleotide variants were mapped onto coding regions using the SNVBox genomic coordinate mapping tool and designated as missense, nonsense, nonstop or silent. Indels were mapped onto RefSeq proteins using the refGene table in the UCSC Genome Browser [50]. Indels were designated inframe, frameshift or splice altering.

### Variant scores

#### VEST classifier training

A Random Forest classifier (ntrees = 1000, mtry = 9) was trained on 47724 missense mutations directly implicated in human inherited disease from the Human Gene Mutation Database (HGMD Professional v2012.2), and 45818 likely neutral missense mutations from the Exome Sequencing Project (ESP6500 accessed 07/2012) using parf Parallel Random Forest software http://code.google.com/p/parf/. Each mutation was described by a vector of 86 quantitative features available through the SNVBox database [51]. HGMD disease mutations were filtered so as to exclude polymorphisms, low-confidence disease mutations and common variants (AF ≥ 1%). This filtering strategy removed 3592 polymorphisms, 4739 low confidence mutations and 492 common variants. An additional 14181 variants that did not cause missense changes were also removed. Twenty-three of the remaining variants did not have a RefSeq accession associated with the variant, 321 consisted of different genomic events that resulted in the identical missense mutation and 47 could not be annotated with features from the SNVBox because the transcript identifier was not supported. ESP6500 mutations were filtered to remove rare variants (AF *<*1%), and any mutations occurring at the same codon as an HGMD disease mutation were dropped. Removing overlap with HGMD resulted in 689823 missense variants, only 46303 of which were present at (AF *<*1%) and mapped onto a RefSeq NM identifier. An additional 485 ESP6500 mutations could not be annotated with features from SNVBox because the transcript identifier was not supported.

#### Feature selection

The training set was partitioned in to 3 approximately equal parts, each with a balanced number of mutations from HGMD and ESP6500. The three partitions were constrained such a that all mutations in the same gene were included in the same partition. We used mutual information between feature values and class labels estimated from the first partition to select all features that provided at least 0.01 bits, 0.001 bits or any positive amount of information about the label. We then trained a VEST classifier using partition 2 and the subset of features selected using partition 1. The 3rd partition was scored with the classifier to determine whether classifier performance improved relative to a classifier trained using all the features.

#### Estimating classifier generalization error

To assess VEST's generalization error, we used 5-fold cross-validation. The VEST training set was divided into 5 partitions such that each partition contained a balanced number of disease and neutral mutations. All mutations occurring in the same gene were constrained to occur in the same partition in order to avoid overly optimistic estimates of the generalization error. We did not constrain homologous genes to occur in the same partition. Receiver Operator Characteristic (ROC) and Precision-Recall (PR) curves were constructed from the predicted class labels and the AUC statistic was used as a measure of classifier performance.

#### Benchmark Construction and Benchmark Variant Scoring with SIFT4.0 and PolyPhen2

For benchmark set construction, we downloaded the PolyPhen2.2.2 training set release dated December 2011 (http://genetics.bwh.harvard.edu/pph2/dokuwiki/downloads). HUGO gene symbols were associated with PolyPhen2 training set variants using a local mirror of the UCSC Genome Browser [52]. The VEST training set was then partitioned into a benchmark test set and a benchmark training set. Any mutation in the VEST training set that occurred in a gene represented in the PolyPhen2 training set was placed into the benchmark training set, and all remaining mutations were placed in the benchmark test set. More HGMD disease mutations overlapped the PolyPhen2 training set than neutral mutations, so additional mutations were removed from the benchmark test partition and placed in the benchmark training partition until a balanced number of each class was reached. To ensure unbiased benchmarking, we moved all mutations in the same gene together such that no gene was split across the benchmark training and test partitions. A VEST classifier was trained on the benchmark training set and used to score the benchmark test set. Genomic coordinates in hg19 for the benchmark test set missense mutations were submitted to the PolyPhen2 batch webserver http://genetics.bwh.harvard.edu/pph2/bgi.shtml as well as to a local installation of SIFT4.0. Since PolyPhen2 offers 2 trained classifiers, HumDiv and HumVar, this procedure was repeated twice.

#### Statistical hypothesis testing framework and empirical null distributions

VEST assigns missense mutations a score between 0 and 1, representing the fraction of decision trees in the Random Forest that voted for the disease mutation class. We used an empirical null model to estimate p-values for each missense mutation based on VEST scores. We constructed three empirical null distributions consisting of CHASM scores for 13639 polymorphisms from the Swissprot variant pages, 28509 variants from the 1000 Genomes Project with AF ≥ 1%, and 3421 high quality variants from the Complete Genomics diversity panel with allele count ≥5in the 69 individuals (Complete Genomics Assembly Software Version 2.0, CGATools version 1.6). P-values for each variant were estimated as the fraction of null distribution VEST scores greater than or equal to the VEST score of the variant. Each empirical null was filtered to remove overlap with the VEST training set. When scoring multiple missense mutations (n *>*10), false discovery rates were estimated using the Benjamini-Hochberg method.

### Gene scores

#### Parametric approaches

We tested two parametric methods commonly used in meta-analysis: Fisher's Method and Stouffer'S Z-score. Both methods require that p-values are uniformly distributed under the null hypothesis. Fisher's method aggregates p-values on the log scale (Eq. 2) such that very small p-values make a larger contribution to the statistic and can be thought of as an 'at least' approach since small p-values will make a larger contribution to the statistic than large p-values. The statistic based on the *k *aggregated p-values is *χ*^2 ^distributed with 2*k *degrees of freedom, providing an overall p-value.

(2) $$X^{2}=-2\overset{k}{\underset{i=1}{\sum }}ln\left(p_{i}\right)$$

Stouffer's Z-score first converts p-values for each variant to the equivalent z-score on the standard normal distribution. These z-scores are then aggregated (Eq. 3) and the cumulative Z-score is used to get a new meta p-value.

(3) $$Z=\frac{\sum_{i=1}^{k}Z_{i}}{\sqrt{k}}$$

#### Truncating mutation scores

Scores for truncating mutations were calculated as 1-allele frequency multiplied by the maximum VEST score for any missense mutation. For this work, four types of mutation were considered truncating: single nucleotide changes resulting in nonsense and nonstop mutations, frameshift mutations, and deletions eliminating a splice site. We used the maximum allele frequency for the variant in the 1000 Genomes dataset, the Exome Sequencing Project European-American dataset or the Exome Sequencing Project African-American dataset.

#### Simulations

We assessed the sensitivity of the gene scoring methods to three parameters: the magnitude of VEST scores for functional mutations, the number of mutations per gene and the fraction of mutations per gene using power simulations. We generated a population of 1000 disease genes by randomly selecting Z-scores from a null distribution (the standard normal distribution) and an alternative distribution (the standard normal distribution with a mean shift) to represent neutral and functional mutations respectively. We used effect size to represent the magnitude of VEST scores. Here, effect size is the distance between the mean of a Z-score distribution for neutral mutations and the mean of a Z-score distribution for functional mutations. If disease mutations receive strong functional scores with VEST, the distances between the means of these distributions will be large, whereas for modestly scoring functional mutations, the distance will be smaller. We tested mean shifts of 0.5. 1.0, 1.5 and 2.0 and varied the number of mutations from 0 to 50. The fraction of mutations sampled from the alternative distribution was varied from 20% to 100% at intervals of 20%. Z-scores sampled for each gene were aggregated according to Stouffer's method, or converted to p-values then aggregated according to Fisher's method, and the resulting statistic was used to assign the gene a p-value. Power was estimated as the fraction of the disease genes that received a p-value *<*0.05. To simulate the effect of variant misclassification on the gene score power to detect disease genes, we simulated genes with misclassification rates based on VEST generalization error estimates. We selected four points along the ROC curve generated using 5-fold gene holdout cross validation of the VEST classifier (TPR = 60%, FPR = 5%; TPR = 70%, FPR = 10%; TPR = 80%, FPR = 15%; TPR = 90%, FPR = 25%)(see Methods: Estimating classifier generalization error).

We tested the sensitivity of our gene scoring method to locus heterogeneity by simulating a set of normal exomes and introducing disease mutations into distinct genes in subsets of the exomes. In order to ensure that simulated exomes reciprocated the variability in mutation count per gene observed in real sequencing data, we used gene-specific distributions of mutation counts from the Freeman Sheldon syndrome and Miller syndrome exomes. Approximately 7000 distinct genes were observed to harbor mutations when all seven real exomes were considered. To simulate a new exome, we sampled a gene-specific mutation count for each of the ~7000 distinct genes and then, for each gene, generated that number of non-disease associated mutations by sampling Z-scores from the standard normal distribution. Next, we selected a gene in each simulated exome to represent a Mendelian disease gene, and sampled an additional Z-score from a mean-shifted standard normal distribution to represent a disease mutation. For recessive cases, two additional mutations were generated per disease gene. Mutation scores were then aggregated for each gene, resulting in a gene level p-value and a gene rank. We ran each simulation 100 times, and varied the number of exomes created, the number of disease genes, and the disease model (dominant versus recessive).

#### Estimating effect size from the VEST training set

In order to estimate the expected effect size for functional mutations versus neutral mutations, we determined Z-score distributions for the disease and neutral classes of the VEST training set using VEST scores acquired by 5-fold gene holdout cross-validation. The 1000 Genomes null model was used to estimate a p-value for each mutation. The p-values were then converted to Z-scores and the distributions for disease and neutral class mutations compared. We found that the distance between the means of the two distributions was 1.85, with the mean of the neutral class Z-scores at 0.12 and the mean of the disease class Z-scores at -1.73.

#### Mendelian gene scores

For each Mendelian disorder dataset, we pooled all variants detected in the exomes, then grouped all variants by gene. Many Mendelian disorders are monogenic, so we removed genes that were not mutated in all case exomes. This assumption can be relaxed to include all genes or genes mutated in at least a subset of disease exomes if locus heterogeneity is suspected. Miller syndrome has previously been observed to follow a recessive inheritance pattern; therefore we also filtered the Miller syndrome data to remove genes that did not show evidence of mutation in both copies in all exomes. Evidence that both copies of a gene were mutated included homozygous mutations, or two heterozygous mutations in the same gene. Where possible, phasing information was used to rule out multiple heterozygous mutations if they occurred in the same copy of the gene.

#### Contrasting gene scores across groups

We used R statistical software to fit a linear model to 1640 genes that were scored in both Freeman Sheldon syndrome and Miller syndrome exomes. Freeman Sheldon syndrome gene p-values generated with the Stouffer Z-score method were used as the dependent variable, and Miller syndrome gene p-values generated with the Stouffer Z-score were used as the independent variable. The residuals resulting from the linear model were used to assess which of the top ranked genes were more likely to result from sequencing artifacts either due to the sequencing technology or because of properties of the reference genome. A large positive residual indicated p-values that were larger in Freeman Sheldon syndrome than would have been expected by the corresponding p-values in the Miller syndrome data while large negative residual indicated p-values in Freeman Sheldon syndrome that were much smaller than would have been expected in Miller data.

## Competing interests

The authors declare that they have no competing interests.

## Authors' contributions

This project was conceived by RK, HC and exome sequences acquired by RK, HC. Exome processing and variant calling performed by CD. HGMD data were contributed by PDS, DNC. VEST classifier and gene score development by HC. Simulations planned and executed by HC, CD. Manuscript was written by HC. All authors read and approved the manuscript.

## Declarations

The publication costs for this article were funded by grants from National Institutes of Health CA 152432 and National Science Foundation DBI 0845275.

This article has been published as part of *BMC Genomics *Volume 14 Supplement 3, 2013: SNP-SIG 2012: Identification and annotation of SNPs in the context of structure, function, and disease. The full contents of the supplement are available online at http://www.biomedcentral.com/bmcgenomics/supplements/14/S3
